# Valedictory Address Delivered before the Graduating Class of the Baltimore College of Dental Surgery

**Published:** 1854-04

**Authors:** W. W. H. Thackston


### ARTICLE IV.

*Valedictory Address delivered before the Graduating Class of the Baltimore College of Dental Surgery.* By W. W. H. THACKSTON, M. D., D. D. S.

GENTLEMEN OF THE GRADUATING CLASS:

As the enlightened traveler stands upon some distant shore, amid the evidences of present, and monuments of past, magnificence; in the blaze of courts and the pomp and pageantry of thrones; surrounded by the ruins of art, whose splendor once untarnished, was the model and pride of ancient genius; but whose faded glories, like the religion and philosophy that consecrated their walls, have been swept in the harvest of time. If some once familiar voice, fresh from the green groves of his far distant home, as he pauses before the most splendid wonders of that stranger clime should break upon his reveries, with what rapturous tumult of feeling would he turn from the scene, though the wreck of cities and empires lay around him to grasp that oft-pressed hand, and hang with delight upon the familiar accents of his native tongue.

The principle of association which so changed the traveller's reveries, and recalled a thousand images of love and affection, carries us back over a few intervening years, and places *us*, no indifferent or reluctant actor, in such a scene as this. The sympathies which you feel, the friendships which you cherish, the lively recollections with which you look upon the incidents of your connexion with your alma mater, and the bright and exultant hopes with which you look upon the theatre of the world; these, these, are no strangers to us. We know, too, the toils and labors of your career, but still we know, that whatever is divine in genius or beautiful in sentiment, whatever is high and splendid in philosophy, have awarded with golden fruit the severities of study, and thrown the colorings of enchantment around the cultivation of science.

And proud as we may be, gentlemen, to renew our connexion with these halls of learning, under circumstances so flattering, we are nevertheless admonished of the difficulty of meeting the expectations it may excite, and we only encounter the occasion because it is associated with some of the freshest hours of our existence—some of the warmest recollections of our youth, with the remembrance of ties, whose moral unity is yet unbroken, and friendships, whose light shines on unextinguished, though brushed by the raven darkness of the wing of time.

It was our good fortune to enjoy the advantages of professional training in this institution, to be in some degree identified with the first workings of a new and untried enterprise; twelve years ago, it was our proud privilege to occupy, with only two others, the position to which you have this evening so honorably attained. Those intervening years have passed swiftly away—we have participated in many of their stirring incidents, and we have experienced our full share of their changes, their sorrows and their joys. But the lapse of years leaves undimmed, the impressions of that hour. We then felt what you now realize, that the crisis had come when we were to put on a new character, that we were thenceforth to take our position among men, and under God, maintain it *as a man*, self dependant and self reliant.

And though we have not subsequently realized all the dreams and aspirations of our youth, though we have not achieved that distinction, or gathered those laurels of triumph which then hung out in luring splendor as incentives to effort, our failure can in nowise be charged to lack of faithful counsel and instruction, to no false principle or philosophy here inculcated, or to any improper example, set before us by the faculty of this school. And when you go hence to-night, you will go feeling, that whatever fate the future may hold in reserve for you, whether you realize your golden visions of wealth, or the nobler reward of just and merited fame, whether it be your future destiny to climb the steep and rugged hill of science, and leave the impress of your genius upon its lofty summit, or whether you perish at its base, "unwept, unhonored and unsung," your alma mater, while sharing in your laurels, and rejoicing in your success, may not be justly reproached with your failure. She has gathered you to her bosom, has nursed you into strength, has taught you to stand and to walk among the votaries of science. She now unfolds her arms, points you the path to usefulness, to fortune and to fame, and bids you go with her blessing upon your heads.

The time was when an occasion like the present would have been most opportune for an "expose" of dental surgery, its claims to be regarded as a science, and the necessity of its recognition as an honorable and useful specialty of the healing art. But happily, gentlemen, that day has gone by. The world now very correctly apprehends the nature and importance of your chosen pursuit, the world is learning, that its duties and requirements are fully up to the capacity of the most gifted and cultivated among men, and believe me, the world is fast becoming as intolerant of dullness and mediocrity in its ranks as once it was of its appeals for caste among the sister sciences.

We shall, therefore, not detain you with a disquisition or analysis of dental science, its divisions and relations, but will ask your attention to a few observations upon the duties and obligations you are now to assume, as practitioners, and as alumni of this institution.

You have enjoyed every advantage and facility that the present age and condition of science affords, to thoroughly fit and prepare yourselves for the important part you have chosen in the drama of life. Your presence and position here to-night abundantly testify how truly you have appreciated, and how well you have improved those advantages. Your blushing honors, and the approving smiles of your friends and preceptors, alike betoken your high resolves, and the energy and fidelity with which you have thus far sought their accomplishment.

But is your education completed? are your efforts to cease with this occasion? have you reached the *ultima thule* of your aspirations? will you go hence, content with the laurels you have already won, and invoke the favors of your profession upon the lone trophies of your young ambition? If so, sad and melancholy will be the fruits of your brilliant accession to its ranks; your course will be fraught with ruin, and your brightest hopes end in disappointment.

Such, however, have not been the lessons you have here learned, and such are not the sentiments and feelings with which you will leave these halls. You have here, if not elsewhere, been taught what education means; that it is not the work of a year, or a series of years, but of a life, devoted to the evolution, development, discipline, and application of *all* our faculties to the objects and exigencies of our calling. And while you have graduated to the honors of this, the first preparatory school known to dental science, you hold in its diploma but the matriculation ticket to the great school of practice, of patient investigation, of untiring research; that school, whose broad domain yet reaches far over rich fields, that bear foliage of perpetual verdure; flowers "that yet bloom and blush unseen;" fruits that stand whitening to an abundant harvest, but no foot-prints of discovery. It is your mission now to explore those untrodden solitudes, to gather those bright flowers of philosophy, those rich fruits of science, and bring them as votive offerings to the altar of your profession.

Science and art, the twin sisters who so beautifully and harmoniously blend their characters and attributes in our calling, are ever growing, ever moving, ever adding to their already full stature and lofty proportions; ever traveling onward and upward towards that perfection that dwells in "light inaccessible." The achievements of to-day are but the finger-posts that point to the discoveries of to-morrow; the triumphs of to-morrow will reveal some hidden truth or develop some richer treasure for its successor.

Science has been eloquently described, "as *not* a pile of building materials where there is contact but no connection, but an edifice vast and magnificent, but unfinished, with its scaffolding still standing and the rubbish of the work still about it, yet an edifice which, when viewed from the vantage-ground of the far-off future, and in the light of the judgment throne, will be seen to have its deep foundations in the eternity of the past, and its culminating dome in the eternity to come." Do you desire to contribute some of the material for this noble and enduring structure? Are you emulous of having your names inscribed in living characters upon its outer walls? Let it then be your firm and abiding resolve to abate not one jot or tittle of your energy in the struggle for excellence and eminence.

From the inherent character of science, and the progressive tendency of the age, you may not safely pause; to loiter by the way, or to stand still, is to retrograde. Let then each day and year be marked by the development of some important truth, or the acquisition of new and valuable resources in your department. You take with the diploma of this school a high moral obligation, so to exercise and cultivate your talents, as that something may thereby be added to the common fund of knowledge. You have here freely and largely availed yourselves of the labors and accumulations of all who have gone before you, and every rule of right, as well as every principle of good morals, demand that you should at least pay interest upon the appropriation. See to it, that you waste not your time upon trifling and ignoble pursuits, lest the "talent you have, be taken away."

You have already learned that there is no "royal road to knowledge," neither is there any easy path to just and honorable fame, "all human works that are good and of value are brought out of labor and difficulty," and the most successful are those who put forth the greatest and most unbending efforts to be *useful* to their fellow men; for after all it is the *usefulness* of any department of science; its capacity to meliorate the condition of man and to minister to his many necessities, that gives to it its value, its dignity and its honor.

Fortunately for you, your lot has been cast upon an age when the usefulness and value of your specialty of the healing art is universally acknowledged; when the facilities for its cultivation are most ample; when the necessities and appreciation of our fellow-men demand the daily ministration of its offices; when its history, though brief, can point to names of worth and merit that would shed lustre upon any pursuit, besides *living* examples of excellence and renown, who have risen in many instances from obscurity, and by the force of genius and energy, have wrought positions and characters that would adorn any vocation of life.

Let these considerations determine you to be close students, as well as industrious practitioners, so long as you exercise the functions, or challenge consideration as dentists.

And this implies that you not only read carefully, and digest thoroughly the literature of your profession proper, that you not only keep posted, in the discoveries and improvements of your specialty, but that you should make the kindred branches of medicine and general surgery, objects of close study and careful investigation. We need not remind you of the intimate relationship which these several departments of science sustain, or tell you that neither can be clearly apprehended, or successfully practiced, without some knowledge of the others.

The physical sciences and the mechanical arts are also subjects of indispensable interest and importance to the dentist. Without the aids derived from these sources, especially the latter, we should be as impotent in practice, as helpless and hopeless in our element, as would the lone mariner, with chart and compass, but without rudder or sail to guide him "o'er the trackless deep."

And yet, to our mortification, not to use the harsher term, disgust, we find, in some few instances, a growing disinclination to be regarded as at all mechanical, a disposition to cast off and disavow those operations ordinarily designated "mechanical operations," or operations in "mechanical dentistry," and that, for no other reason that we can perceive, save that ignorant and vulgar pride, has ever looked with scornful eye and upturned nose upon every species of labor as in some degree degrading.

But we need not pause to vindicate the respectability of the mechanic arts, we need not make an argument to convince you that whatever contributes in equal degree to man's comfort and happiness, is equally respectable, equally honorable, whether elaborated in the silent chambers of thought, or forged by the brawny hand of toil. And let me impress the truth that it is your religious duty to avail yourselves of, and cultivate all the resources and appliances that Providence has or may place at your disposal, to make your profession in the largest degree a boon, to suffering, decaying, and dying humanity, nor deem yourselves degraded by any honorable agency to accomplish good.

In a word, gentlemen, we would have you ambitious, ambitious of distinction as cultivated virtuous men of science, ambitious as ministering servants to the ills and woes of flesh, not emulous of fame alone, for what is mere fame ? It cannot ward off the calamities of life, or postpone the hour of death, the loudest notes of human applause reach not the cold and silent precincts of the grave.

Not of wealth, for "riches take to themselves wings and fly away," but we would have you emulous of that CHARACTER among your fellow men, founded upon the exercise of cultivated minds, the illustration of highest excellence in your profession, and the exhibition of the moral virtues and christian graces in your daily intercourse with society.

And here permit me to observe, that a proper reverence for that Being whose eye kindles the fires, and whose hand controls the motions of the universe, is the basis of all moral, as well as religious, principle. It subdues our passions, moderates our tempers, exalts our conceptions, and ennobles our impulses. It spreads the pathway of existence along valleys green with perennial verdure, and flings o'er the short twilight of life a portion of that immortality which is dawning beyond the tomb.

This is the distinction of which we would have you emulous, this the fame, if you please, of which the truly great have a foretaste in this life, and which carries with it the consolation "that to live in hearts they leave behind, is not to die." These are the influences that will incite you to noble deeds, that shall cause your names, if not written in the pages of history, to be recorded in the memory of your fellow men, in characters so indelible that they shall stand the changes of time, and fade from remembrance only when its cycles shall cease to be told.

But, gentlemen, this is not a period in your career to be marked by mere passive musings and speculations, the tide of human events is not to drift you from these halls to a vast solitude, where you may pursue in learned leisure scholastic and scientific abstractions. But you are about to be called into contact and sympathy, with that energy and activity which marks the age in which we live. You are to begin the work for which you have here sought preparation, you are now to try your strength with the more stern realities of life; enter into the struggle with brave hearts and willing hands. Let not indolence eat away your precious hours, let not the leaden wing of sloth cast its blighting shadow upon your pathway.

"Wide is the field of your usefulness, varied and important the duties of your office; it may not be your vocation to keep sad vigil beside the couch of death, to count the ebbing strokes of life's failing tide, to utter the fearful words, which like ice-bolts rive the heart, or to speak the blessed accents of hope that clothe the pale countenance of the midnight watcher with smiles;" yet in the sphere of your duty the responsibility is relatively great. The issues of life and death do not often hang upon your operations, yet they sometimes do—but if this were never so, still there is yet enough of happiness or pain involved, to keep alive that just sense of responsibility and earnest solicitude with which you should ever approach their performance.

Carry into your professional practice all the kindlier sympathies of your nature; be gentle, patient and forbearing with those who ask your assistance; seek to assuage their sorrows, and mollify the stern ministrations of your art by amiable and considerate deportment, yet always be firm in the exercise of your best judgment, yield not to the shrinking of the timid and weak, make no compromise with the dictates of ignorance or caprice, conduct every operation in accordance with your convictions of the right and proper way—and if this be not submitted to or acquiesced in, why politely decline the case. It is better to lose an ignorant or whimsical patron than to forfeit your self-respect, better to hurt your pocket than stab your reputation.

As your professional and social relations must bring you in contact with the cultivated and refined, be ever mindful of your appearance and deportment. We believe it was Chesterfield, who pronounced a tasteful dress and polished manner the best letter of introduction, and rest assured that to dentists this maxim applies with peculiar force. Nothing will more readily inspire respect, or more warmly attach your patients than scrupulous neatness and attention to personal appearance, combined with graceful and easy manners.

Let it not be supposed, however, that in advocating the cultivation of our social powers, we countenance the contemptible littleness of dandyism. The mere dandy or fop we despise as a thing whose proper definition the great American philologist and lexicographer has given in the following appropriate terms, "a male, of the human species, who dresses himself like a doll, and carries his character on his back." Between the peculiarities of such a creature and the dignified suavity and refinement of the educated gentleman, it were odious to institute a comparison.

A modest, dignified, unostentatious bearing, will render you most acceptable with all grades and conditions of society, arrogance and noisy pompous pretension are not held as evidences of worth, but are rather regarded as indications of conscious inferiority. "*Esse quam videri malim,*" is a safe and sound maxim, which you would do well to remember in all the relations of life.

In your intercourse with the profession, be always kind, frank and fraternal; a generous and lofty ambition may make you rivals, but can never make you enemies. Render cheerfully any service or assistance in your power to a brother in difficulty; communicate freely, whatever it may be your good fortune to originate or discover, not alone for the love you bear him, not alone for the good of science, but for the respect you cherish for yourself; for he who conceals or appropriates any valuable discovery or invention in science, sullies his own good name with a stain that no time can efface.

With the unworthy of the profession, the ignorant pretender, while you can hold no fellowship, yet, by a kind word of counsel and encouragement, which you can well afford to bestow, you may show him "a more excellent way." But if you find him incorrigible and firmly joined to his idols, let him alone. Quacks in every department of the healing art, pettifoggers in law, and impostors in religion, have always infested society, and always will, until the ignorance and credulity upon which they grow and flourish, is lost in the light of milennial glory. And as vain and futile will be the *attempt* at their correction or extinction, while the elements of their reproduction and sustenance exists, as to change the laws of gravitation or arrest the march of pestilence, without correcting the miasm in which it breeds.

We have thus, gentlemen, imperfectly sketched some of the more important obligations to science, to society, to your professional brotherhood and to yourselves, which you have assumed as dentists. And, in conclusion, we wish to utter a few reflections upon the obligations you owe as graduates and alumni of the Baltimore College of Dental Surgery.

When you have left these scenes and associations, to enter upon your respective fields of labor, when you begin to taste the sweets of professional success, and to realize the fruits of your well directed efforts, remember your alma mater, and that you have here left those who have toiled with you, and for you, who will cherish a lively interest in your advancement and promotion; who will ever feel in your individual success and happiness the most anxious solicitude, who have committed to you their own good name, and no unimportant agency in their own ultimate destiny. We believe that you will prove worthy the confidence reposed in you, and that you will preserve intact the honors with which they have vested you.

Twelve years ago, we found that it was something to be the accredited alumnus of the first and then the *only* college of dental surgery, and that, too, in its infancy. The result of the experiment which it then was esteemed, its utility and permanency, were to be determined by its fruits. It was then something to be the representative of its teachings, and the bearer of its diploma. But how much greater matter is it now? now that its permanent existence and necessity have become fixed facts? now that the utility of the scheme has been demonstrated, and its policy generally approved? now that its diploma will at once command respect, and inspire confidence throughout the civilized world, how shall we estimate the worth of our alma mater, and what limits can we fix to the obligation which her maternal care and far-reaching influence has imposed?

The establishment of this institution, and its maintenance, has cost years of self-sacrificing toil and anxious solicitude, and one would think, in contemplating the intrinsic worth of the scheme, the patient and unremitting effort which has been applied to its successful prosecution, and the rich and satisfactory results which have already been realized; that it was an enterprize that would enjoy the approval and good will of all men. But no, it offers no exception to the general rule, that nothing in this world, however good, or however great, can claim exemption from the assaults of wickedness and folly.

Esau, gentlemen, though a somewhat notable example, and representative of an unfortunate class, has, notwithstanding, had successors and disciples in every succeeding age, who have ever stood ready to sacrifice their richest treasures to the gratification of unholy appetites and unhallowed passions, and our own profession, in this enlightened day, can furnish examples of wanton recklessness, that would barter our rich heritage for a "mess of pottage," that would, for a paltry and selfish gratification, despoil our temple, overturn its altars, and shiver its shrines.

It is a duty imposed upon you by every sentiment of gratitude, of patriotism, and of chivalry, to stand by your college, and the system which it illustrates, to guard its courts against the goths and vandals of the nineteenth century, to strengthen its foundations, and add to its graceful proportions.

Let us be true to ourselves, true to the trust committed to us.

"We are but few, a little band,
Be faithful till we die,
Shoulder to shoulder let us stand,
Till side by side we lie."

Let us show ourselves worthy our high birthright, and the Baltimore College of Dental Surgery will hold, through time, its position as first and noblest of its kind. It may pass, (as it has already done,) through hours of darkness and trial, the storms of calumny and traduction may beat upon it, envy and malice may pour upon it the venom of their thousand tongues. But it will stand sure and steadfast, an imperishable monument of the wisdom and benevolence of its founders, of their concern for the promotion of science and the welfare of men.

"As some tall cliff that lifts its awful form,
Swells thro' the vale and midway leaves the storm,
Though round its breast, the rolling clouds are spread,
Eternal sunshine settles on its head."

*Gentlemen of the Graduating Class*—Our part in the exercises of this occasion is accomplished. It only remains for us, as a representative of the profession you have chosen, to extend to you the right hand of fellowship, and a cordial welcome to its ranks. May your success in life be commensurate with your merits, and may your merits shed undying lustre upon your names. On behalf of the Faculty of this school, we bid you farewell—not in the sadness and gloom of a final and hopeless separation, but in the joyous trust, that we shall meet you again—bearing the rich fruitage of well spent years.

